# In Theory and in Practice

**Published:** 1920-11-13

**Authors:** 


### COLOUR BLINDNESS

### In Theory and in Practice.

When we consider that as many as 4 per cent. of men are to a greater or lesser degree deficient in their ability to distinguish colours, it is a matter for astonishment that the condition known as colour blindness was described little more than 100 years ago. The reason no doubt is that the sufferer does not realise his condition; those around him talk of reds and greens, purples and violets, or blues and indigos, and it escapes him that while the difference between blue and indigo is nothing, between purple and violet little more than a matter of opinion, that between red and green is to the normal man a fundamental difference. Also, should he venture a question on this subject he is mocked, which also tends to prevent the condition becoming known.

### Sex Incidence.

We have spoken of the sufferer as "he" with reason; women seldom seem to be colour-blind, not, that is, in the medical sense, though artistically it is only too common. But, though she escapes herself, the condition tends to be transmitted by the daughter of a colour-blind father to her sons. Many interesting family histories have shown the fact.

Dalton, the founder of modern chemistry, in the last years of the eighteenth century was the first to describe the condition from which he himself suffered, and since his time facts have been accumulated. To explain them many theories have been elaborated, but the grasping of the really vital points and their significance is of very recent date. After a long struggle against complacent ignorance on the part of Authority, Dr. Edridge-Green has at last succeeded in placing the subject on a scientific basis, which has resulted in the entire reorganisation of the tests for colour-blindness employed in the Navy, the mercantile marine, and on railways. Not only so, but, as so often happens, the study of this pathological condition has thrown great light on the normal, and he is now able to put forward a theory of vision, far more convincing that any of its predecessors.

The basis of all colour work is the spectrum of white light which gives to most of us seven distinct colours—red, orange, yellow, green, blue, indigo, and violet. Many cannot distinguish indigo between the blue and the violet; these are classed as hexa-chromics, and are only to be detected by means of careful analysis. The next class, the penta-chromics, cannot distinguish the orange from the red and the yellow, but they are not likely to cause any trouble at sea or on the railway, nor are the tetra-chromics to whom blue blends either with the green or the violet.

When we get to the tri-chromics, who see yellow as a red-green, we reach the danger-zone, for yellow lights are common, and a colour which can be described as red-green is a very dangerous combination for an engine driver to see at night. The di-chromics see red and violet, and obviously are entirely unsuitable either on the footplate of a locomotive or at sea. There have been recorded cases who saw in one colour alone, but these are rare.

The colour test in vogue until the general adoption of the Edridge-Green tests was the Holmgren wool test, named after the Swedish scientist. In this test the method employed was to match various skeins of wool, but not to name the various colours. Now persons lacking colour vision have frequently developed their sense of tone to an extraordinary extent; they may not know a green from a red, both being to them different shades of one colour, but if asked to match a definite skein of, say, green wool they will with the greatest accuracy choose a perfect match, not to the colour, but to the intensity of colour, in fact the tone.

A railwayman is not expected to match the colours of the signal lamps, but to recognise them as red or green as the case may be. Therefore, if the naming of colours is substituted for the matching of colours in the wool test, something is gained, but even then there is the difficulty that the person tested can compare the particular skein with the others and gain much help from so doing. Also, the tone of reds is different from the tone of greens. In proof of this, look at the lamps on any signal gantry; in fine weather the green is much brighter than the red, while in foggy weather the red will show up when the green is quite invisible.

### The Testing Lantern.

In the Edridge-Green lantern the various bands of the spectrum can be exposed to the view of the person to be tested; he can be shown any single colour, or combination of colours, or sequence of colours, and the intensity of these colours can be varied at will.

The intensity of the colour is a very important factor, for as a normal person watches the spectrum while the light is slowly decreased he passes through all the stages of colour blindness. First, indigo, should he be able to distinguish it, goes, then orange, then blue, then yellow, then green; finally, a twilight grey is all that is left.

As to the theory of vision which is now put forward, it still has many blanks which require to be explained by such terms as "vital action," which means "we know it happens, but we don't know how." Roughly, Dr. Edridge-Green would have us to see with our "cones" alone, the "rods" in the retina act as secretory cells for the visual purple, which bathes and activates the cones, this action only taking place in the presence of light.

A well-known fact supports this theory. The *fovea centralis*, the area of most acute vision,

* There is a poster advertisement now prominent in London in which a rainbow is shown across a breaking wave: in this rainbow spectrum, the blue is actually put next to the red—possibly the artist is colour blind?—Ed.

**Colour Blindness**—(*continued*).

contains only cones. If in the dusk one stares at a small bright light, say a lamp or a star, the light after a short time disappears. Now the image of the light fell on the fovea alone. In the effort required to perceive the light visual purple was used, and finally used up, and, most important of all, as no light fell on any rods no more purple was liberated, hence the cones of the fovea became insensitive.

Again, kinematograph pictures might be expected to be more successful in a dark room. However, the contrary is the case, for in a fairly light room the whole retina is made active and plenty of visual purple is produced; whereas in a darkened room the chief illumination is on the fovea, and little on the crowded rods at the periphery of the retina, resulting in a shortage of purple for the cones to see by.

Night blindness, when the patient is quite blind in weak lights, is well explained this way. It is an acknowledged deficiency disease and is associated with a condition of *retinitis pigmentosa*. Doubtless the starved rods develop an excess of pigment, just as other inefficient organs undergo a primary stage of hypertrophy, but need a greater stimulus to free the activating substance for the benefit of the cones than is afforded by the weak light of the late evening. Nor do a few bright lights help, as they could only be seen by the fovea, and it is short of purple, whereas a lantern near by cures the condition, its rays falling on the rods as well as the central cones promoting the formation of the visual purple.

